# Insight toward the MicroRNA Profiling of Laryngeal Cancers: Biological Role and Clinical Impact

**DOI:** 10.3390/ijms21103693

**Published:** 2020-05-24

**Authors:** Takashi Takeuchi, Hiromichi Kawasaki, Amalia Luce, Alessia Maria Cossu, Gabriella Misso, Marianna Scrima, Marco Bocchetti, Filippo Ricciardiello, Michele Caraglia, Silvia Zappavigna

**Affiliations:** 1 Department of Precision Medicine, University of Campania “Luigi Vanvitelli”, 80138 Naples, Italy; takashi.takeuchi@unicampania.it (T.T.); Hiromichi.KAWASAKI@unicampania.it (H.K.); amalia.luce@unicampania.it (A.L.); alessiamaria.cossu@biogem.it (A.M.C.); gabriella.misso@unicampania.it (G.M.); marco.bocchetti@unicampania.it (M.B.); silvia.zappavigna@unicampania.it (S.Z.); 2 Molecular Diagnostics Division, Wakunaga Pharmaceutical Co., Ltd., Hiroshima 739-1195, Japan; 3 Drug Discovery Laboratory, Wakunaga Pharmaceutical Co., Ltd., Hiroshima 739-1195, Japan; 4 Biogem Scarl, Institute of Genetic Research, Laboratory of Molecular and Precision Oncology, 83031 Ariano Irpino, Italy; marianna.scrima@biogem.it; 5 Division of Otorhinolaryngology, “A. Cardarelli” Hospital, 80131 Naples, Italy; filipporicciardiello@virgilio.it

**Keywords:** microRNA, biomarkers, head and neck cancer, laryngeal cancer, prediction, prognosis, metastasis, lifestyle habit, chemo-/radio resistance, therapeutic target

## Abstract

Head and neck squamous cell carcinoma (HNSCC), a heterogeneous disease arising from various anatomical locations including the larynx, is a leading cause of death worldwide. Despite advances in multimodality treatment, the overall survival rate of the disease is still largely dismal. Early and accurate diagnosis of HNSCC is urgently demanded in order to prevent cancer progression and to improve the quality of the patient’s life. Recently, microRNAs (miRNAs), a family of small non-coding RNAs, have been widely reported as new robust tools for prediction, diagnosis, prognosis, and therapeutic approaches of human diseases. Abnormally expressed miRNAs are strongly associated with cancer development, resistance to chemo-/radiotherapy, and metastatic potential through targeting a large variety of genes. In this review, we summarize on the recent reports that emphasize the pivotal biological roles of miRNAs in regulating carcinogenesis of HNSCC, particularly laryngeal cancer. In more detail, we report the characterized miRNAs with an evident either oncogenic or tumor suppressive role in the cancers. In addition, we also focus on the correlation between miRNA deregulation and clinical relevance in cancer patients. On the basis of intriguing findings, the study of miRNAs will provide a new great opportunity to access better clinical management of the malignancies.

## 1. Introduction

Head and neck squamous cell carcinoma (HNSCC) represents the sixth most common cancer in the world, and is a histologically and genetically heterogeneous disease. It arises from multiple anatomical sites including oral mucosa, tongue, salivary glands, nasopharyngeal, pharyngeal, and laryngeal carcinomas [1,2]. Despite substantial progress in both treatment strategies and diagnostic techniques, the overall survival rate as well as the mortality rate of the disease is still largely unimproved over the last decades [3,4]. Smoking tobacco and drinking excessive alcohol are the predominant risk factors of initiation and aggressive progression of HNSCC [2,5]. 

Both local and distant metastases usually result in poor prognosis of HNSCC, thus representing one of the most adverse phenomena in the patients [6].

The survival rate of HNSCC patients is still poor since the disease is often at advanced stages when diagnosed. Thus, early and precise detection in the initial and pre-clinical stages of the tumor is crucial to achieve high survival rate of HNSCC patients, but the early diagnosis is often hampered by present lack of definite symptoms. Implementation of early intervention is greatly dependent on clinical judgment by diagnostic instruments, histological results, and usable markers. However, there is still no truly reliable and identifiable tools with sufficient sensitivity and specificity at the initial stages. To overcome the issue, easily and universally available protocols have been long investigated in order to prevent initial and secondary malignant tumors. For example, many biomolecule markers, such as proteins, DNA, and RNA molecules, have been studied but there is still a big challenge to use them as a certain biomarker for the clinic purpose in HNSCC patients. Another issue, protocols for therapeutic clinical management, have not been yet established to accurately diagnose the presence of small metastases that cannot be evidenced by conventional radio-imaging analysis. Additionally, there is currently a lack of information on clinically available molecular-based biomarkers for both prediction and prognosis of aggressive malignancies in a non-invasive way. In fact, a misleading of false positive/negative metastases cannot be completely avoided, resulting in expanding tumor invasion (under treatment) or undergoing an unnecessary surgical intervention (over treatment). Hence, it is necessary to urgently address these issues by either early prediction and detection indicators or improved therapeutic treatments.

**Figure 1:**
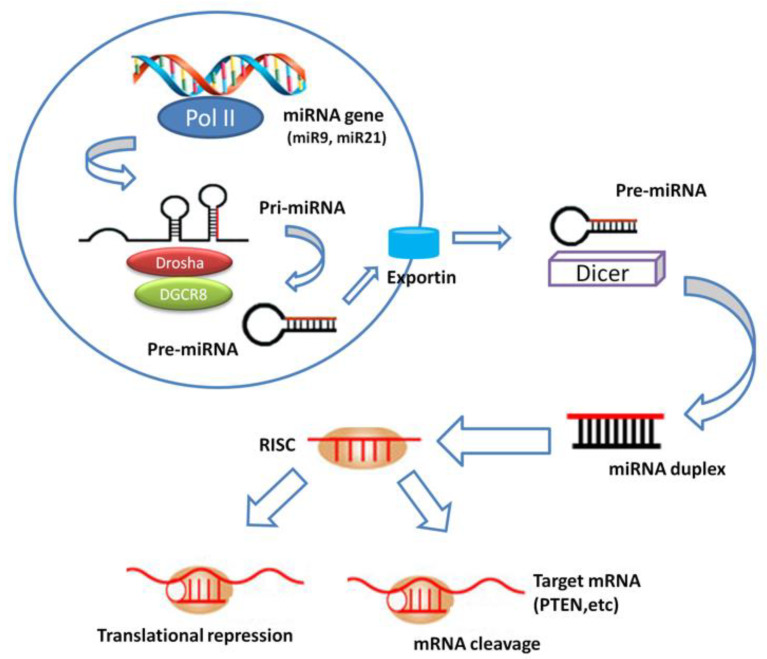
MiRNA biogenesis. miRNA gene is transcribed to generate a primary miRNA (pri-miRNA) precursor molecule that undergoes nuclear cleavage to form a second precursor miRNA (pre-miRNA). The pre-miRNA is exported in the cytoplasm and cleaved to generate a microRNA duplex containing the mature miRNA. The duplex unwinds and the mature miRNA assembles into RISC. The miRNA base-pairs with target mRNA to perform gene silencing trough mRNA cleavage or translation repression.

Recently, miRNAs have emerged as robust predictors and prognosticators, pharmaceutical drugs, personalized medicine, and therapeutic biomolecular targets for the effective treatment of diseases. This small group of highly conserved non-protein coding RNAs (approximately 20 nucleotides in length) is essential for maintaining physiological conditions. miRNAs negatively and post-transcriptionally regulate divergent genes involved in (signal) transduction pathways, through promotion of mRNA destabilization or prevention of protein translation machinery by either perfect or nearly perfect binding to the complementary site of mRNA at 3′ untranslated regions [7]. Biogenesis of miRNAs has been currently studied and summarized in many reviews, but not fully elucidated due to its complicated procedures. Conventionally, biogenesis pathway involves two cleavage steps, one nuclear and one cytoplasmic (Figure 1), but other alternative biogenesis pathways that require a different number of cleavage events and key enzymes are possible. The mechanism by which microRNA precursors are sorted to the different pathways is unclear but probably depends on the origin site of the microRNA, its sequence and thermodynamic stability. However, mature miRNAs usually exhibit a tissue-specific pattern of expression but an apparent tissue-specific pattern for their corresponding primary transcripts has not been found [7,8,9]. Consequently, miRNAs may act either as onco-promotors or tumor suppressor genes, depending on their biological targets and the cellular context [10]. Numerous studies have indicated that miRNA dysregulation patterns could be useful for prediction of clinical outcome in HNSCC patients. 

In recent years, investigations of circulating extracellular miRNAs have rapidly progressed. Although miRNAs were thought to be unstable in extracellular environment due to their low stability, it has been confirmed the presence of a stable form of circulating miRNAs in human body fluids including blood, saliva, and urine [11,12,13,14,15,16,17]. Among patients, the amount of circulating miRNAs is also changed and closely linked to pathological events including metastases. There are several advantages of circulating miRNAs including easy and safe accessibility, ready detection and quantization, and good reproducibility. It is likely that circulating miRNAs are suitable as an informative application for the diagnostic examination and are able to monitor the real-time disease status by detecting their specific signatures. Here, we briefly summarize recent miRNA studies regarding biological functions, comprehensive expression profiles, and clinical relevance in HNSCC patients, particularly focused on laryngeal carcinoma.

## 2. miRNA Deregulation 

### 2.1. Laryngeal Cancer

Laryngeal cancer (LCa) is one of the most common types of neoplasms in the head and neck region and accounts for approximately 1–2% of all malignancies. Over 90% of LCa is histologically classified as squamous cell carcinoma. LCa is divided into glottis (60–65%), supraglottic (30–35%), or subglottic carcinoma (1–2%). Cigarette smoking, and heavy alcohol consumptions are the most detrimental risks of the disease [18,19,20,21]. In addition, it has been reported that the presence of nodal metastases is the most significant prognostic factor of LCa patients. Prediction of the overall survival rate in LCa patients is more related to node metastasis than tumor extension [22]. Metastases are often responsible for the aggressiveness and cancer-related mortality. Partial laryngectomy, chemo-radiation, and combination therapy are effective treatments of laryngeal carcinomas at an early clinical stage. However, in the advanced cases of LCa patients, total laryngectomy is often required as a therapeutic intervention, but such surgical modality strongly negatively affects patient’s quality of life. Furthermore, cancer recurrence may appear at the laryngeal region or other distant sites even though all parts of the malignant larynx were correctly removed by the laryngectomy. Therefore, it is highly and urgently demanded to enhance conventional methods and to set up both more effective treatment management and earlier nodal metastasis prediction techniques in order to reduce cancer morbidity and mortality and to improve the quality of life. Regarding the diagnostic approaches, one of the possible ways is the establishment of new reliable biomarkers to predict LCa progression and to prognosticate the disease. As mentioned above, many researchers have recently focused on miRNAs as an excellent tool in the biological, clinical, and medical field. So far, a number of research groups have widely investigated that miRNA dysregulation, which is up/down expression, is observed in LCa when comparing to precancerous lesions, benign, or noncancerous counterparts, using microarray or quantitative real-time PCR (qRT-PCR) analysis.

Several miRNAs are aberrantly upregulated in LCa and they can contribute to aggressiveness of the cancer. MiR-9 was upregulated in LCa tissues and cell lines by influencing malignant development at the larynx through direct regulation of PTEN [23].

miR-10b is overexpressed in LCa, showing a role in the initiation of cancer invasion and migration in the laryngeal carcinoma cell line Hep-2. Zhang et al. unveiled that miR-10b accelerates the epithelial-mesenchymal transition (EMT) in Hep-2 cells through directly targeting E-cadherin (E-cad). The authors studied both epithelial (E-cad and ZO-1) and mesenchymal (Vim, N-cad, and FN) markers in Hep-2 cells with ectopic miR-10b expression using western blot analysis, and they showed that miR-10b-transfected cells possess reduced epithelial and increased mesenchymal markers. The regulation of CDH1 (E-Cad gene) by miR-10b was also confirmed in this study [24].

miR-21 is one of the most well-studied miRNAs and exerts oncogenic roles in different types of malignancies. It was exhibited that miR-21 is upregulated in laryngeal tumor tissues and its inhibition by antago-miRs caused growth inhibition of Hep-2 cells via the suppression of BTG2, a validated miR-21 target, leading to inhibition of cell cycle progression without influencing cell apoptosis. Other targets of miR-21, PTEN, TPM1 and PDCD4, were also downregulated [25].

Additionally, a repressor of cell cycle progression, the cyclin-dependent kinase 2-associated protein 1 (CDK2AP1), which is a G1/S transition inhibitor, was found to be a negative regulator of LCa. CDK2AP1 was reported as a target gene of miR-21 in pathological human oral keratinocytes [26] and a recent investigation showed that this gene was downregulated by miR-205, indicating that miR-205 regulates CDK2AP1 in LCa. Hence, miR-205 works as an oncogene, through the suppression of CDK2AP1, and affects cell proliferation and motility by promoting MMP2 and MMP9 activities and both c-Myc and CyclinD1 over-expression in LCa cells [27].

Overexpression of miR-93 was found in LCa tissues by global miRNA screening and was confirmed by qRT-PCR set [28].

Another manuscript reported that miR-93 was inversely correlated with cyclin G2 (CCNG2) levels in clinical samples from LCa patients. A gain of function analysis showed that miR-93 promotes cell proliferation and metastases, while suppression of miR-93 reduces these malignant processes. Further studies revealed that miR-93 accelerates cancer progression in LCa through directly inhibiting CCNG2 [29].

Plasma miR-126 was associated with clinical differentiation of LCa. The authors also showed that miR-126 in part regulated calmodulin-regulated spectrin-associated protein 1 (Camsap1), and the introduction of miR-126 mimic decreased LCa tumorigenesis in xenograft mouse models [30].

Lian and coworkers found that miR-132 was strongly overexpressed in LCa tissues and cells and directly targeted FOXO1, which is a class of human forkhead box O (FOXO) proteins and works as important effectors of PI3K/Akt signaling. This study thus demonstrated the oncogenic role of miR-132 in LCa by mediating PI3K/AKT/FOXO1 pathway [31].

miR-221 was identified as an oncogene through the modulation of several signaling pathways. Inhibition of endogenous miR-221 expression reduced cell proliferation and induced both apoptosis and cell cycle arrest of LCa cells. In addition, the study of miR-221 in LCa also showed to stimulate apoptosis resistance by affecting apoptotic protease activating factor-1 (Apaf-1). Interestingly, miR-221 inhibitor had anti-cancer effects as confirmed in xenograft mouse models inoculated with Hep-2 cells, showing that miR-221 suppression led to reduced tumor size and weights and to increased survival rate [32].

Additionally, overexpression of miR-302-3p was found in LCa tissues and cells and disclosed to be involved in EMT processes. The authors identified that tumor repressor Smad4 is a direct downstream target of miR-302-3p [33].

Guan and coworkers observed an up-regulation of miR-423-3p in primary LCa cell lines. The same group also confirmed that miR-423-3p plays an oncogenic role. In silico prediction algorithms and further validation confirmed that adiponectin receptor 2 (AdipoR2) is directly regulated by miR-423-3p [34].

Several reports exhibited lower miRNA expression in LCa, and the miRNAs could suppress tumor development and metastasis through multiple direct/indirect target oncogenes.

Many studies have recently shown that miR-1 is often downregulated in various tumors and is an anticancer gene. miR-1 downregulation and its clinical impact were also found in HNSCC specimens including LCa.

It was reported that miR-1 is significantly under-expressed in LCa tissues and ectopic miR-1 expression induces the suppression of cell growth and metastatic potential in Hep-2 cells. Target identification assay showed that miR-1 directly regulates fibronectin 1 (FN1), which has cancer metastasis related functions. Thus miR-1 exerts an anti-oncologic role in LCa [25,35].

miR-24 was significantly underexpressed in LCa tissues or cell lines compared to paired normal tissues or normal human keratinocyte cell lines. Reintroduction of miR-24 inhibited both cell growth and colony formation and promoted apoptosis. The research group revealed that miR-24 binds to the 3′-UTR of XIAP mRNA [36].

A well-known ubiquitous onco-suppressive miRNA, miR-34, is frequently downregulated in a variety of cancers [37].

miR-34a was inversely correlated to cyclin D1 (CCND1) levels, nodal metastases, and clinical stage in LCa. It was also found that miR-34a regulates CCND1 gene [38]. miR-34c is also downregulated in LCa and functions as a tumor suppressor. Replacement of miR-34c expression in LCa cells induced both inhibition of growth and invasion by directly targeting c-Met [39].

Another study in Hep-2 cells demonstrated that miR-34a/c directly regulates UDP-*N*-acetyl-α-d-galactosamine:polypeptide-*N*-acetylgalactosaminyltransferase 7 (GALNT7) a member of glycosyltransferases involved in cancer spreading and metastasization [40].

miR-144 expression was found to be downregulated in LCa, and to have anti-oncogenic functions through the suppression of insulin receptor substrate 1 (IRS1). The latter stimulates the phosphatidylinositol 3-kinase (PI3K)/Akt pathway that is then inhibited by miR-144 [41].

miR-340 is another anti-oncogene in LCa facilitating p27 expression and blocking PI3K/Akt signaling through the suppression of the histone methyltransferase EZH2, thus resulting in the inhibition of cell proliferation, metastatic abilities, and apoptosis [42].

The association between Let-7a downregulation and high-mobility group protein A2 (HMGA2) mRNA overexpression in LCa indicates that it is involved in tumor aggressiveness and metastasis. HMGA2 is able to change the DNA structure through its interaction, thus affecting DNA transcription. The expression levels of Let-7a and HMGA2 were strongly associated with clinical stage, differentiation grade, and lymph node metastases in LCa [43].

Taken together, these reports open the new opportunity to use miRNAs as either biomarkers or therapeutic approaches for patients affected by LCa. 

### 2.2. Other HNSCC

Deregulation of miRNAs in other HNSCC, such as oral squamous cell cancer (OSCC), tongue, and salivary gland tumors [44,45,46,47,48,49,50,51,52,53,54,55,56,57,58,59,60,61,62,63,64,65,66,67,68,69,70,71,72,73,74,75,76,77,78,79,80,81,82,83,84,85,86,87,88,89,90,91,92,93,94,95,96,97,98,99,100,101,102,103,104,105,106,107,108,109,110,111,112,113,114,115,116,117,118,119,120,121,122,123,124,125,126,127,128,129,130,131,132,133,134,135,136,137,138,139,140,141,142,143,144,145,146,147,148] is summarized in Table 1. Unfortunately, data demonstrating the role of miRNAs in the other HNSCCs are still not sufficient to provide an accurate overview. miR-155 was found to be upregulated in OSCC tissues and its overexpression reduced the intracellular levels of cell division cycle 73 (CDC73), a target of a tumor suppressor gene [47]. Another study also reported miR-155 overexpression in OSCC cells and tissues [48]. Upregulated miR-155-5p induced cancer metastases to neck lymph nodes and was associated with poor overall survival rate in OSCC patients. Inhibition of miR-155-5p promoted the epithelial marker E-cadherin, reduced signal transducer and activator of transcription 3 (STAT3), and activated suppressor of cytokine signaling 1 (SOCS1) in an OSCC cell line [49,50]. 

Liu et al. showed that miR-222 expression was downregulated in oral tongue squamous cell carcinoma (OTSCC) harboring highly metastatic potential compared to the lower invasive one. Ectopic expression of miR-222 led to a decrease in cell invasive ability and reduced both Matrix Metallopeptidase 1 (MMP1) and Superoxide Dismutase 2 (SOD2) expressions in OTSCC, through direct regulation of the mRNAs [61].

Furthermore, in tongue cancer tissues, miR-26a was also downregulated in comparison with paired non-pathological ones. Restoration of miR-26a showed inhibition of cell proliferation and apoptosis induction, suggesting that miR-26a elicits anti-cancer effects in tongue carcinogenesis. Further functional studies showed that miR-26a inhibited directly DNA methyltransferase 3b (DNMT3B) transcript [72]. Another report showed downregulation of miR-26a and miR-26b in tongue squamous cell carcinoma (TSCC) tissues. In addition, overexpression of these miRNAs suppressed TSCC cell cycle, motility and glycolysis, while enhanced cell apoptosis by direct interaction with p21 Activated Kinase 1 (PAK1) [73].

Another group showed that miR-140-5p was decreased in hypopharyngeal squamous cell carcinoma (HSCC) tissues, and the downregulation was correlated with tumor size and lymph node metastases. Moreover, functional analysis showed that miR-140-5p directly suppresses ADAM10. This study demonstrated that miR-140-5p suppressed cancer invasion and migration by inhibiting ADAM10-mediated Notch1 signaling pathway [85]. 

Either oncogenic or tumor suppressive miRNAs and their validated target genes in LCa and other HNSCCs are reported in Table 1. 

Overall, likewise the knowledge in LCa, these recent articles also provide a rationale for developing miRNA-based therapeutic weapons or understanding molecular mechanisms underlying of HNSCC biology.

## 3. miRNAs as Biomarkers for the Prediction of Initiation and Progression

### 3.1. Laryngeal Cancer

Increasing evidence has suggested the usefulness of miRNA signatures as robust predictors for early and accurate diagnosis of LCa. A number of research groups have currently shown unique and attractive candidate miRNAs as biomolecule-based markers.

A research group showed that the combination of exosomal serum miR-21 and long non-coding RNA HOTAIR exhibits high sensitivity (94.2%) and specificity (73.5%), respectively, in different malignancies from benign laryngeal disease, thereby indicating that the combination may be suitable for diagnosis of LCa [94].

Another study also reported miR-21 as a potent prediction tool. MiR-21 upregulation and miR-375 downregulation were observed in LCa samples. Interestingly, the ratio of miR-21/miR-375 expression had a robust value with great sensitivity (94%) and specificity (94%) for the prediction of LCa [95].

A study by Saito et al. showed that miR-133b, miR-455-5p, and miR-196a were abnormally expressed in laryngeal tumors if compared to their matched non-oncological tissues. Subsequent validation tests confirmed that miR-196a was over-expressed in cancer tissues against precancerous dysplasias and laryngeal benign tissues. Moreover, in situ hybridization confirmed specific expression of miR-196a in both cancer and cancer stroma cells [96].

In early laryngeal carcinoma and normal esophageal mucosa tissues, microarray-based screening showed two highly deregulated miRNAs, hsa-miR-657 and hsa-miR-1287, with good specificity and sensitivity to classify laryngeal malignancies at early stages [97].

A report showed that both miR-148a and miR-375 levels were highly upregulated in LCa tissues. Association analysis unveiled that miR-375 expression in advanced LCa (stage III/IV) was far greater than in tumors at early stage (stage I/II). On the contrary, despite increased miR-148a level in early cancer tissues, its expression was not significantly changed when comparing to advanced ones [98].

To our knowledge, Ayaz et al. have carried out the first study on circulating miRNA profiling in plasma collected from LCa patients. In this research, several circulating miRNAs were found in the liquid biopsies and abnormally expressed across plasma specimens if compared to control plasma. Five circulating miRNAs (miR-212-3p, miR-331-3p, miR-603, miR-660-5p, and miR-1303) were detectable in the oncological plasma, but they were not detected in plasma taken from healthy individuals or patients presenting with any other diseases [99].

Serum miR-378 was more highly expressed in LCa patients than healthy controls, and the expression was significantly decreased after surgical intervention. Also, miR-378 expression was correlated with clinical stage, but did not associate to tumor size [100].

In a recent study, the expression levels of serum miRNAs in LCa patients were investigated using microarray screening and furthermore validated by qRT-PCR. The results showed eight up-regulations (miR-31, miR-33, miR-141, miR-149a, miR-182, LET-7a, miR-4853p and miR-122) and three down-regulations (miR-133a, miR-145 and miR-223) in LCa patients (*n* = 66) compared to healthy volunteers (*n* = 100). Moreover, ROC curve analysis indicated that miR-31, miR-33, and LET-7a possessed high diagnostic ability for the disease with AUC (Area under curve) = 1.0 [101]. In addition, several miRNAs reviewed on the above part could be potential diagnostic markers of LCa. To summarize, these miRNAs could contribute to the development of novel predictive and diagnostic biomarkers of LCa.

### 3.2. Other HNSCC

Evidence has also suggested the usefulness of miRNA signatures as biomarkers for the prediction of initiation and progression of other HNSCCs [102,103,104,105,106] but reports referring to the other HNSCCs are still limited. A miRNA expression ratio of miR-221:375 showed great discriminatory potential, with great sensitivity (92%) and specificity (93%) between HNSCC tissues and non-pathological ones [104].

In the investigation of circulating miRNAs in oral cancer specimens, both circulating miR-196a and miR-196b were significantly overexpressed in plasma obtained from patients with oral pre-cancer lesions and in plasma from oral cancer patients. Both miRNAs showed high discriminative values between pre-cancer patients and normal, and between cancer patients and normal samples. Furthermore, the combined determination of miR-196a and miR-196b possesses high sensitivity (91%) and specificity (85%) in predicting potential malignancy at early stages [105].

## 4. miRNAs as Biomarkers for the Prognosis 

Despite the improved therapeutic options of the past few decades, the treatment of HNSCC and the patient’s quality of life are still unsatisfying due to poor prognosis of HNSCC. The deregulated miRNA expression patterns can also help clinicians to prognosticate cancer progression and outcome. Table 2 also showed several reported miRNAs, which could be useful as prognostic biomarkers of LCa and other types of HNSCC.

### 4.1. Laryngeal Cancer

As reported above, LCa patients with higher miR-21 or lower miR-375 levels in cancer tissues showed poorer prognostic values than those with low miR-21 or high miR-375 levels [95].

miR-23a expression was up-regulated in LCa tissues when comparing to their corresponding normal ones; additionally, miR-23a overexpression was related to lymph node metastases and the five-year survival rate of patients. Further biological analysis showed that up-regulation of miR-23a facilitated cancer migration and invasion [107]. 

Strikingly, miR-101 downregulation was closely associated with nodal involvement, tumor grade (T3-T4), and LCa at advanced stage. Moreover, clinical relevance was observed between lower miR-101 level and poor outcome. Ectopic miR-101 overexpression reduced cell proliferation and migration and led to cell-cycle arrest and apoptosis. Besides, miR-101 suppressed tumor growth in a xenograft model mouse with LCa, thereby suggesting that miR-101 acts as a tumor suppressor gene [108]. 

Another report showed that the expression of miR-9 was higher in LCa if compared to the paired normal laryngeal tissues, and the study also reported a relation between miR-9 overexpression and poorer prognosis of LCa [109]. 

The article showed that miR-296-5p was correlated to tumor relapse in LCa at an early stage [110].

Cappellesso et al. found a significant downregulation of miR-200a and miR-200c levels in LCa from the group of patients who developed disease recurrence [111].

Collectively, these findings suggest that miRNAs could be useful as potential biomarkers to prognosticate the clinical outcomes.

### 4.2. Other HNSCC

As shown in Table 2, several recently reported miRNAs in HNSCC can be useful as predictors and/or prognosticators [112,113,114,115,116,117,118] even if data referring to the potential role of miRNAs in other HNSCCs are still not sufficient to provide an accurate overview.

A decrease in miR-218, miR-125b, or Let-7g expression is associated with advanced clinical stage and lymph node metastasis and is a useful prognostic factor of OSCC patients [113].

High miR-93 expression was correlated with T classification, clinical stage, lymph node involvement, and poor prognosis among HNSCC patients [28,115]. In addition, elevated serum miR-93 was found and associated with prognosis of NPC patients.

Bufalino et al. reported both miR-143 and miR-145 downregulation and high activin A levels in OSCC cell lines and tissue specimens. Downregulation of a miR-143/miR-145 cluster controlled cancer invasiveness and was clinically correlated to nodal metastasis and worse survival [116].

Serum miR-9 level was significantly lower in patients with OSCC or oral leukoplakia in comparison to that of non-pathological controls, and low serum miR-9 expression was correlated to poor overall survival and disease-free survival rate [118].

## 5. Resistance Related miRNAs 

The resistance is one of the most important determinants of prognosis and treatment results. Resistance to chemotherapy or irradiation often blocks the effective therapeutic modality of malignant tumors, resulting in further cancer development, invasiveness, recurrence, and consequent unfavorable outcomes. Recent findings have suggested that miRNAs are closely associated with radio/chemo-resistance in human cancers through complex processes controlling huge number of genes in cell signaling network; therefore, the use of miRNAs can become a significant breakthrough to improve cancer treatment protocols. 

### 5.1. Radio-Resistance

An overall miRNA profiling assay showed that 4 miRNAs, miR-296-5p, miR-452, miR-183*, and miR-200c, were aberrantly altered in LCa tissues taken from radio-resistant patients. Importantly, a subsequent validation set exhibited that miR-296-5p was linked to resistance to radiation [110].

Suh et al. reported that miR-196a overexpression enhanced the radio-resistance in HNSCC cells [119].

An investigation using available HNSCC samples in the cancer genome atlas (TCGA) miRNA database identified that a 5 miRNA signature (downregulated let-7e and upregulated miR-16, miR-29b, miR-150, and miR-1254) could be useful for the prediction of radiation responsiveness in HNSCC patients. Additionally, this study also indicated that higher levels of ataxia-telangiectasia mutated expression (ATM) in HNSCC patients were correlated to increased resistance to radiotherapy [120].

De Jong et al. reported a comprehensive miRNA expression profiling correlated to radio-resistance in HNSCC cells from primary LCa. Validation studies showed that reduced expression of miR-203 was correlated to local recurrence after radiation and resistance to irradiation in LCa patients. On the other hand, overexpression of miR-203 reduced EMT processes [121].

It was reported that miR-138-2-3p and miR-218 in HNSCC were associated to radio-sensitivity as revealed by two independent studies [122,123].

Ahmad et al. demonstrated that the expression levels of miR-15b-5p in HNSCC patients, treated by definitive intensity-modulated radiotherapy, were significantly high for long time of locoregional control (LRC) compared to short time of LRC. Moreover, Kaplan-Meier and multivariable Cox regression analyses showed that miR-15b-5p could be a useful as a prediction marker of radiotherapy response for the disease [124].

### 5.2. Chemo-Resistance

Yin and co-workers identified multiple drug resistance (MDR)-related miRNAs in drug resistant LCa cells, suggesting that miRNAs can help clinicians to predict chemo-sensitivity and to design a therapeutic strategy to overcome drug resistance in LCa [125].

Likewise, miR-181a was found to inhibit Twist1 mediating EMT, enhancing metastatic potential, and inducing cisplatin chemo-resistance in TSCC cells. In this context, Twist1 was confirmed as a direct target gene of miR-181a [126].

miR-222 downregulation by antisense transfection enhanced the sensitivity of OSCC to cisplatin through directly blocking PUMA (p53-upregulated modulator of apoptosis) gene expression, and thereby offering a combination of miR-222 antisense and cisplatin as a new powerful therapeutic approach [127].

miR-101-3p repressed cell proliferation and metastatic functions and induced apoptosis in a salivary gland adenoid cystic carcinoma cell (ACC) line. The authors also showed that miR-101-3p directly regulated Moloney murine leukemia virus 1 (Pim-1) oncogene and promoted the sensitivity to cisplatin in ACC cell lines [128].

Qin et al. revealed that exosomal miR-196b originated from cancer-associated fibroblasts conferred cisplatin resistance in head and neck cancer (HNC) cells through targeting CDKN1B and ING5. In addition, high expression of exosomal miR-196b in plasma was clinically correlated with poor overall survival and chemoresistance, suggesting the miRNA as a prediction biomarker and a therapeutic target for cisplatin resistance [129]. In Table 3 combinatorial studies between miRNAs and radio/chemotherapy are summarized. 

### 5.3. Thermal-Resistance

Hyperthermia is a potential therapeutic regimen for treating various cancers by damaging and killing tumor cells induced by high temperature heating and is generally employed as combination therapy with an established method, especially radiation and/or chemotherapeutic approach. A major advantage of hyperthermal treatment is tolerable for patients due to minimal damage to normal tissues and very few or no severe toxic effects. 

A unique study showed an interesting association between miRNAs and thermal resistance in OSCC. Comparing miRNA levels in thermal-sensitive OSCC cells against resistant cells, 5 miRNAs (downregulated miR-23a and miR-27a, upregulated miR-30a, miR-30c, and miR-203) were differentially regulated. Notably, reintroduction of miR-27a in resistant cells significantly accelerated hyperthermia-induced cell death and inhibited HSP90 and HSP110 expressions, implying that miR-27a was positively associated with thermal sensitivity through HSPs modulation [130]. 

miR-218 regulation can be also associated to thermal-chemotherapy in gastric cancer [131]. 

These studies are informative for a further understanding to control thermal treatment combined with the conventional remedies. Furthermore, this relatively new research field, which improves the efficacy of hyperthermal therapy through simultaneously targeting or replacing miRNAs as potential adjuvant therapy, may progress rapidly and deeply as an emerging attractive therapeutic approach in the future.

As summarized in Table 4, we reviewed previous papers studying miRNAs in thermo/radio/chemo-sensitivity in HNSCC.

## 6. Infection and Life Habit Related miRNAs 

### 6.1. HPV Infection

Human papillomavirus (HPV) has been known as the main etiological cause of cervical cancer. HPV infection has also negative impacts on HNSCC patients. Among HNSCCs, it has been known that oropharyngeal carcinoma is more commonly related to HPV status. HPV-positive head and neck cancer is classified into a distinct category in comparison to the typical HNSCC because of its different histopathological and clinical parameters. However, HNSCC with the infection is still poorly characterized despite accumulating knowledge. Moreover, the virus infection alters normal miRNA expression patterns. The identification of HPV-related miRNA deregulation gives us the informative knowledge to more clearly understand the changes of underlying biological and pathological molecular processes induced by the virus and to improve clinical management and prognostic outcome. Herein we focused on the effect of HPV status on miRNA deregulation patterns in HNSCC [132,133,134,135,136,137,138].

Lajer et al. described that miRNA expression patterns were associated to HPV status in HNSCC patients. Their study showed that miR-15a/miR-16/miR-195/miR-497 family, miR-143/miR-145, and miR-106-363 cluster may play crucial roles in the pathogenesis of HPV [133].

Gao et al. demonstrated that the deregulation of 6 miRNAs was associated to cancer survival in oropharyngeal SCC patients. Three miRNAs (miR-142-3p, miR-146a, and miR-26b) were upregulated in surviving patients, while the remaining miRNAs (miR-31, miR-24, and miR-193b) were upregulated in patients who died. Moreover, 5 HPV-associated miRNAs (miR-9, miR-223, miR-31, miR-18a, and miR-155) signatures were identified in this investigation [135]. More recently, a combination of 5 miRNAs (let-7g-3p, miR-6508-5p, miR-210-5p, miR-4306, and miR-7161-39) was also shown to correlate with the survival rate of HPV-negative HNSCC patients [136]. 

Bersani et al., showed that overexpression of miR-155 in tonsillar and base of tongue cancer (TSCC/BOTSCC) was associated with HPV positivity and improved survival, while low miR-185 expression associated with HPV negativity and decreased survival for the disease. ROC curve analysis with combination of these miRNAs exhibited prognostic ability for survival with AUC of 0.71, suggesting their usefulness as prognostic markers and therapeutic targets [138]. However, reports about the role of miRNAs in the other HNSCCs, particularly, in the complex scenario of HPV-related OPSCCs are still limited.

### 6.2. Smoking Tobacco and Alcoholic Beverage

As described above, smoking tobacco products and consuming excess alcoholic drinks negatively influence miRNA expression, leading to their deregulation, and subsequently promote the pathogenesis and progression of tumors through the regulation of many signaling pathways. The study of miRNA aberration by the use of tobacco or alcohol is mainly focused on and reported in oral cavity, trachea, and either lung or liver regions [139,140]. In this session, we described recent reports investigating a relationship between miRNAs and the life habit.

Manikandan et al. revealed that high miR-155 levels in OSCC patients are associated with the habit of tobacco/betel quid chewing [141].

To investigate the negative impact on tobacco constituents in oral fibroblasts, Pal et al. studied abnormal miRNA alterations using oral fibroblast models exposed to cigarette smoking. As a result, miR-145 downregulation in response to smoking exposure was observed in the model [142]. This result is supported by a manuscript which exhibited a decrease of miR-145 expression in lungs of a mouse model exposed to cigarette smoke [143]. 

A previous report showed that miR-133a-3p was underexpressed in oropharyngeal squamous cell carcinoma tissues originated from HPV(+) smoker patients in comparison with that ones from HPV(+) non-smokers. Moreover, the downregulation of miR-133a-3p was also confirmed both in serum and metastatic lymph nodes in the same study. Further examination revealed that reduced miR-133-3p increased both EGFR and HuR mRNA expression which may lead to HPV- associated cancer progression [144].

Gong et al. demonstrated that high expression of miR-499a was associated with lower overall survival and N stage in high tobacco exposed HNSCC patients [145].

In healthy individuals, miRNA patterns are also changed by the use of smoking [146,147]. These results suggest that smoking affects both tissue miRNAand circulating miRNA profiles, resulting in pathological events.

Normal oral keratinocytes were treated with biologically relevant doses of ethanol and acetaldehyde to examine which miRNAs were related to alcohol intake. RNA-sequencing analysis identified significant upregulation of eight miRNAs in alcohol-associated HNSCC. Among them, miR-30a and miR-934 were the most significantly overexpressed miRNAs and were also confirmed by qRT-PCR. On the basis of these results, alcohol components induced strong miR-30a and miR-934 upregulation, which may cause alcohol-associated oncological events [148].

Members of miR-34 family, miR-34a and miR-34c-5p, are also clinically identified as alcohol-associated miRNAs in HNSCC [74,149].

miR-183 overexpression was associated to high alcohol intake in tongue cancer patients [150].

A comprehensive study showed the deregulation of 4 miRNAs (miR-101, 181b, miR-486, and miR-1301) in epithelial cells (HaCaT and OKF4) exposed to cigarette treatment, indicating their involvement in smoking-related HNSCC development [151].

Collectively, these miRNAs represent a new evidence to better understand molecular biology of HNSCC and its correlation with lifestyle (Table 5).

## 7. Conclusions and Perspectives

MicroRNAs have recently emerged as great potentials for both biomarkers and therapeutic targets of human diseases including cancers. In this review, we briefly summarized biological roles, miRNA aberration, and relationship between microRNA deregulation and clinical relevance in HNSCC. Several previous studies have found deregulation of miRNAs expression in different types of HNSCC and explored their use as potential biomarkers for cancer detection and/or prognosis. Single and combinatorial miRNA in silico analysis revealed that miRNAs dysregulated targeted genes and pathways that are involved in cancers [71,152].

Although microarray-based screening assay has a limitation, which is that the evaluable number of miRNAs is restricted to the immobilized probes for miRNA sequences on the platform and is impossible to discover a novel miRNA, the technique has been widely used so far for the assessment of deregulated miRNAs. Initial microarray and subsequent validation analysis is currently the gold standard to determine miRNA expression pattern. Several well-studied miRNAs, such as miR-1, miR-21, and miR-34, were also dramatically up/down deregulated in HNSCC, thereby clearly showing that these miRNAs are importantly responsible for cancer aggressiveness or repression through their target genes. Recent researchers have also unveiled the stable presence of circulating miRNAs in liquid biopsies taken from HNSCC patients. Findings about circulating extracellular miRNAs in systemic circulation are gradually and steady accumulated, and these miRNAs have remarkable potential as minimally invasive molecule-based markers for the prediction of node metastases and response to therapy, diagnosis, and prognosis of HNSCC. In this light, our group performed a multicentric study to identify a specific miRNA expression profile for laryngeal cancer. We found 20 miRNAs specific for laryngeal cancer and a tissue-specific miRNA signature that consists of 11 miRNAs, seven of which are upregulated and four downregulated, predictive of lymph node metastases [153]. These results are enormously innovative, at least in our opinion, and lead to the definition of a group of potential specific biomarkers for LCa that will allow to improve its early diagnosis and to identify patients with minimal residual disease or recurrence; moreover, they can be used to predict prognosis in patients that show the specific miRNA signature suggestive for nodal involvement. In this case, the miRNAs will be useful to select a tailored treatment.

This review also described a correlation between resistance to cancer therapies and change of miRNA levels. These data are useful for the prediction of the response to radio/chemotherapy and help clinicians to select the best therapeutic approaches for patients.

Lifestyle habits, such as consuming smoke products and excess alcohol, may promote HNSCC through disruption of normal miRNA regulation, resulting in poor prognosis. Accumulating evidences may provide a deeper understanding of pathological mechanisms in cancers and advise us to reconsider our lifestyle in order to maintain healthy life.

Despite advances in the research of miRNAs, there are some controversies deciphering the biological function of a miRNA, which is either tumor suppressive or oncogenic action even in the same category of cancer. One of the major reasons is tumor heterogeneity, largely contributing to the complication of interpretation mainly due to different gene expression levels in each cancer cell. The discrepant roles of miRNAs in HNSCC revealed by earlier studies could be explained by the differences of pre-analytical factors, sample collection methods, storage conditions, RNA extraction, platforms for the examination, the number of evaluated samples, selection of the control for data normalization, and the used statistical analysis. In addition, blood-derived miRNAs released by tumor cells and their deregulation patterns are not frequently consistent with those in (tumor) tissues. This could be due to the presence of the contaminated miRNAs by other components derived from circulating normal cells, lysed or apoptotic cells, or other malignant tissues, across the tested liquid samples. In fact, it is difficult to correctly clarify the origin of circulating miRNAs. Hemolysis and above-described both clinical and experimental variations also influence circulating miRNA profiles, eventually giving us conflicting findings. Therefore, establishment of standardized protocols, which eliminates variability across each sample and is commonly usable in laboratories and clinics, is essential for the study of miRNAs for diagnostic and therapeutic purposes prior to the use of clinical settings. Recent reports suggest that multiple miRNA-based profiles have higher diagnostic, prognostic and therapeutic performance as well as higher sensitivity than individual miRNA assays because the combination of different miRNAs, that regulate multiple target genes, can better explain how each of them contributes to carcinogenesis and better represent the comprehensive biological effect of miRNA regulation in the multistep process leading to cancer. Furthermore, miRNA signatures, consisting of a plurality of different miRNAs, allow to better distinguish between different pathologies while single miRNAs alone are frequently not disease-specific. On the other hand, single miRNA markers are often verified by independent studies, miRNA signatures are less frequently validated. With an increasing number of validated miRNA signatures and with the advance of matured high-throughput approaches in clinical settings, specific miRNA markers are likely to contribute to human healthcare. All of these reported miRNAs have a high possibility to successfully reach the clinical setting as either diagnostic tools or therapeutic targets of these malignancies. We believe that miRNAs will be readily available as robust applications for HNSCC patients in the next future.

## Figures and Tables

**Table 1:** miRNA deregulation in head and neck cancer and validated targets.

Cancer Type	miRNA	Regulation	Role	Target Gene	Ref.
Laryngeal cancer (LCa)	Let-7a	Down	Tumor-suppressive	*HMGA2*	[43]
	miR-1	Down	Tumor-suppressive	*FN1*	[25,35]
	miR-9	Up	Oncogenic	*PTEN*	[23]
	miR-9	Up	Oncogenic	*-*	[109]
	miR-10b	Up	Oncogenic	*CDH1*	[24]
	miR-21	Up	Oncogenic	*BTG2, PTEN, TPM1, PDCD4, CDK2AP1*	[25]
	miR-23a	Up	Oncogenic	*-*	[107]
	miR-24	Down	Tumor-suppressive	*XIAP*	[36]
	miR-34a	Down	Tumor-suppressive	*CCND1, GALNT7*	[38,40]
	miR-34c	Down	Tumor-suppressive	*c-Met, GALNT7*	[39,40]
	miR-93	Up	Oncogenic	*CCNG2*	[28,29]
	miR-101	Down	Tumor-suppressive	*CDK8*	[108]
	miR-126	-	Tumor-suppressive	*Camsap1*	[30]
	miR-132	Up	Oncogenic	*FOXO1*	[31]
	miR-138-2-3p	Up	Oncogenic	*-*	[122]
	miR-144	Down	Tumor-suppressive	*IRS1*	[41]
	miR-203	Down	Tumor-suppressive	*-*	[121]
	miR-205	Up	Oncogenic	*CDK2AP1*	[27]
	miR-221	-	Oncogenic	*ER-α, p27, p57, c-kit, Apaf-1*	[32]
	miR-296-5p	Up	Oncogenic	*-*	[110]
	miR-302-3p	Up	Oncogenic	*Smad4*	[33]
	miR-340	Down	Tumor-suppressive	*p27, EZH2*	[42]
	miR-423-3p	Up	Oncogenic	*AdipoR2*	[34]
Head and neck squamous cell carcinoma (HNSCC)	miR-1	Down	Tumor-suppressive	*TAGLN2*	[65]
	miR-1	Down	Tumor-suppressive	*EGFR, c-Met*	[70]
	miR-10b	Down	Tumor-suppressive	*-*	[45]
	miR-99	Down	Tumor-suppressive	*IGF1R, mTOR, Akt1*	[76,77]
	miR-100	Down	Tumor-suppressive	*IGF1R, mTOR, Akt1*	[76,77]
	miR-101	Down	Tumor-suppressive	*EZH2, rap1GAP*	[81,82]
	miR-146a	Down	Tumor-suppressive	*-*	[50]
	miR-155	Down	Tumor-suppressive	*-*	[50]
	miR-196a	Up	Oncogenic	*-*	[45]
	miR-203	Up	Oncogenic	*-*	[54]
	miR-203	Down	Tumor-suppressive	*-*	[56]
	miR-204	Down	Tumor-suppressive	*Brd4*	[91]
	miR-205	Up	Oncogenic	*-*	[54]
	miR-206	Down	Tumor-suppressive	*EGFR, c-Met*	[70]
Oral squamous cell cancer (OSCC)	miR-1	Down	Tumor-suppressive	*Slug*	[68]
	miR-1-3p	Down	Tumor-suppressive	*DKK1*	[69]
	miR-26a/b	Down	Tumor-suppressive	*TMEM184B*	[71]
	miR-34a	Down	Tumor-suppressive	*-*	[74]
	miR-99-3b	Down	Tumor-suppressive	*glycogen synthase kinase-3β*	[80]
	miR-99a	Down	Tumor-suppressive	*Myotubularin-related protein 3*	[78,79]
	miR-104-5p	Down	Tumor-suppressive	*PAK4*	[86]
	miR-155	Up	Oncogenic	*-*	[141]
	miR-155	Up	Oncogenic	*CDC73*	[47,48,49]
	miR-181a	Down	Tumor-suppressive	*K-ras*	[52]
	miR-181a/b	Up	Oncogenic	*-*	[51]
	miR-204	Down	Tumor-suppressive	*Sox4, Slug*	[88]
	miR-204-5p	Down	Tumor-suppressive	*CXCR4*	[89]
	miR-222	Up	Oncogenic	*PUMA*	[60]
	miR-222	Down	Tumor-suppressive	*PUMA*	[127]
	miR-223	Up	Oncogenic	*-*	[62]
	miR-223	Up	Oncogenic	*FBXW7*	[63]
	miR-494	Down	Tumor-suppressive	*HOXA10*	[90]
Oral	miR-10b	Up	Oncogenic	*-*	[44]
Oral tongue squamous cell carcinoma (OTSCC)	miR-222	Down	Tumor-suppressive	*MMP1, SOD2*	[61]
Hypopharyngeal squamous cell carcinoma (HSCC)	miR-140-5p	Down	Tumor-suppressive	*ADAM10*	[85]
Maxillary sinus	miR-1	Down	Tumor-suppressive	*TAGLN2, PNP*	[66]
Nasopharyngeal carcinoma (NPC)	miR-1	Down	Tumor-suppressive	*ET-1*	[67]
	miR-10b	Down	Tumor-suppressive	*-*	[46]
	miR-101	Down	Tumor-suppressive	*ITGA3*	[83]
	miR-204	Down	Tumor-suppressive	*Cdc42*	[90]
	miR-494-3p	Up	Oncogenic	*Sox7*	[93]
Salivary adenoid cystic carcinoma (SACC)	miR-104-5p	Down	Tumor-suppressive	*Survivin*	[87]
	miR-181a	Down	Tumor-suppressive	*MAP2K1, MAPK1, Snai2*	[53]
Salivary gland adenoid cystic carcinoma (SGACC)	miR-101-3p	Down	Tumor-suppressive	*Pim-1*	[128]
Sinonasal squamous cell carcinomas (SN-SCC)	miR-34a	Down	Tumor-suppressive	*BCL-2*	[75]
Tongue	miR-21	Up	Oncogenic	*-*	[148]
	miR-183	Up	Oncogenic	*-*	[148]
Tongue squamous cell carcinoma (TSCC)	miR-26a	Down	Tumor-suppressive	*DNMT3B*	[72]
	miR-26a	Down	Tumor-suppressive	*PAK1*	[73]
	miR-26b	Down	Tumor-suppressive	*PAK1*	[73]
	miR-140-5p	-	Tumor-suppressive	*LAMC1, HDAC7, PAX6*	[84]
	miR-181a	Down	Tumor-suppressive	*Twist1*	[126]

**Table 2:** Potential predictive and/or prognostic biomarker in HNSCC.

miRNA	Regulation	Cancer	Diagnosis/Prognosis	Ref.
Let-7a	Up	LCa	Diagnosis	[101]
Let-7g	Up	OSCC	Prognosis	[113]
miR-100	Down	HNSCC	Prognosis	[117]
miR-101	Down	LCa	Prognosis	[108]
miR-10b	Up	OSCC	Diagnosis	[44]
miR-125b	Up	OSCC	Prognosis	[113]
miR-125b	Down	HNSCC	Prognosis	[117]
miR-1287	Down	LCa	Diagnosis	[97]
miR-1303	Up	LCa	Diagnosis	[99]
miR-133b	Down	LCa	Diagnosis	[96]
miR-143	Down	OSCC	Prognosis	[116]
miR-145	Down	OSCC	Prognosis	[116]
miR-146a	Down	HNSCC	Diagnosis	[50]
miR-155	Up	OSCC	Prognosis	[48,49]
miR-15a	Down	HNSCC	Diagnosis, Prognosis	[117]
miR-181a	Down	SACC, TSCC	Prognosis	[53,126]
miR-196a	Up	LCa, OSCC	Diagnosis	[96,105]
miR-196b	Up	OSCC	Diagnosis	[105]
miR-199b	Down	HNSCC	Diagnosis, Prognosis	[117]
miR-200a	Down	LCa	Prognosis	[111]
miR-200c	Down	LCa	Prognosis	[111]
miR-203	Up	HNSCC	Prognosis	[54]
miR-203	Down	HNSCC	Prognosis	[121]
miR-204	Down	OSCC	Prognosis	[88]
miR-205	Down	HNSCC	Prognosis	[102]
miR-205	Up	HNSCC	Prognosis	[112]
miR-205-5p	Down	OSCC	Diagnosis, Prognosis	[103]
miR-21	Up	HNSCC	Prognosis	[114,117]
miR-21	Up	LCa	Diagnosis, Prognostic	[94,95]
miR-212-3p	Up	LCa	Diagnosis	[99]
miR-218	Up	OSCC	Prognosis	[113]
miR-223	Up	HNSCC	Prognosis	[114]
miR-23a	Up	LCa	Prognosis	[107]
miR-296-5p	Up	LCa	Prognosis	[110]
miR-31	Up	LCa	Diagnosis	[101]
miR-33	Up	LCa	Diagnosis	[101]
miR-331-3p	Up	LCa	Diagnosis	[99]
miR-34c	Down	HNSCC	Diagnosis, Prognosis	[117]
miR-375	Down	LCa	Diagnosis, Prognosis	[95]
miR-375	Down	LCa	Diagnosis	[98]
miR-378	Up	LCa	Diagnosis	[100]
miR-455-5p	Up	LCa	Diagnosis	[96]
miR-603	Up	LCa	Diagnosis	[99]
miR-657	Up	LCa	Diagnosis	[97]
miR-9	Up	LCa	Prognosis	[109]
miR-9	Down	OSCC	Prognosis	[118]
miR-93	Up	HNSCC	Prognosis	[28,115]
miR-99a	Down	HNSCC	Prognosis	[114]

HNSCC: Head and neck squamous cell carcinoma; LCa: Laryngeal cancer; OSCC: Oral squamous cell cancer; SACC: Salivary adenoid cystic carcinoma; TSCC: Tongue squamous cell carcinoma.

**Table 3:** Combinatorial studies between miRNAs and chemio/radiotherapy.

miRNA	Cancer	Sample Type	Combinatorial Treatment	Effect	Ref.
miR-24	LCa	LSCC cell lines (Hep-2, AMC-HN-8)	Overexpressed miR-24 (pGCMV/miR-24) + Radiation	Enhanced radiosensitivity: Suppresses cell proliferation and induces cell apoptosis	[36]
miR-196a	HNSCC	HNC cell line (HN30)	miR-196a knockdown (miR-196a sponge plasmid) + Radiation	Enhanced radiosensitivity: Decreases cell viability	[119]
miR-138-2-3p	LCa	LCa stem cells (originated from Hep-2)	Overexpressed miR-138-2-3p (100 nM miR-138-2-3p mimic) + Radiation	Enhanced radiosensitivity: Inhibits cell proliferation, viability, invasion and induces cell apoptosis, cell cycle arrest and DNA damage	[122]
miR-218	Oral cancer	Oral cancer stem cells	Overexpressed miR-218 (pLV-miR-218) + Radiation	Enhanced radiosensitivity: Decreases cell viability	[123]
miR-181a	TSCC	DDP-resistant TSCC cell line (originated from CAL27)	Overexpressed miR-181a (miR-181a mimic) + Cisplatin	Enhanced chemosensitivity: Decreases IC_50_ value to cisplatin	[126]
miR-222	OSCC	OSCC cell line (UM1)	miR-222 knockdown (anti-miR-222) + Cisplatin	Enhanced chemosensitivity: Induces cell apoptosis by up-regulation of pro-apoptotic PUMA expression and reduces cell invasiveness and IC_50_ value to cisplatin	[127]
miR-101-3p	SGACC	SGACC cell lines (SACC-LM, SACC-83)	miR-101-3p knockdown (anti-miR-101-3p) + Cisplatin	Enhanced chemosensitivity: Inhibits cell growth and induces cell apoptosis	[128]
miR-196a	HNC	HNC cell line (CAL27)	miR-196a knockdown (anti-miR-196a) + Cisplatin	Enhanced chemosensitivity: Promotes cell apoptosis and decreases colony formation	[129]

LSCC: Laryngeal squamous cell carcinoma; HNSCC: Head and neck squamous cell carcinoma; LCa: Laryngeal cancer; OSCC: Oral squamous cell cancer; SACC: Salivary adenoid cystic carcinoma; TSCC: Tongue squamous cell carcinoma SGACC: Salivary gland adenoid cystic carcinoma; HNC: head and neck cancer; DDP: cisplatin.

**Table 4:** Relationship between miRNA deregulation and resistance in HNSCC.

miRNA	Regulation	Cancer	Resistance	Target Gene	Ref.
let-7e	Down	HNSCC	Radiation	*-*	[120]
miR-101-3p	Down	SGACC	Chemotherapy	*Pim-1*	[128]
miR-1254	Up	HNSCC	Radiation	*-*	[120]
miR-138-2-3p	Up	LCa	Radiation	*-*	[122]
miR-150	Up	HNSCC	Radiation	*-*	[120]
miR-16	Up	HNSCC	Radiation	*-*	[120]
miR-181a	Down	TSCC	Chemotherapy	*Twist1*	[126]
miR-183-star	Up	LCa	Radiation	*-*	[110]
miR-196a	Up	HNSCC	Radiation	*Annexin A1*	[119]
miR-196b	Up	HNC	Chemotherapy	*CDKN1B, ING5*	[129]
miR-200c	Up	LCa	Radiation	*-*	[110]
miR-203	Down	LCa	Radiation	*-*	[121]
miR-203	Down	OSCC	Thermotherapy	*-*	[130]
miR-210	Up	LCa	Chemotherapy	*-*	[125]
miR-218	-	OSCC	Radiation	*Bim1*	[123]
miR-222	Down	OSCC	Chemotherapy	*PUMA*	[127]
miR-23a	Up	OSCC	Thermotherapy	*-*	[130]
miR-24	Down	LCa	Radiation	*XIAP*	[36]
miR-25-star	Down	LCa	Chemotherapy	*-*	[125]
miR-27a	Up	OSCC	Thermotherapy	*-*	[130]
miR-296-5p	Up	LCa	Radiation	*-*	[110]
miR-29b	Up	HNSCC	Radiation	*-*	[120]
miR-30a	Down	OSCC	Thermotherapy	*-*	[130]
miR-30c	Down	OSCC	Thermotherapy	*-*	[130]
miR-424	Down	LCa	Chemotherapy	*-*	[125]
miR-452	Up	LCa	Radiation	*-*	[110]
miR-494	Down	LCa	Chemotherapy	*-*	[125]
miR-923	Up	LCa	Chemotherapy	*-*	[125]
miR-93	Down	LCa	Chemotherapy	*-*	[125]
miR-93-star	Down	LCa	Chemotherapy	*-*	[125]

**Table 5:** HPV, smoking, or alcohol related miRNA deregulation.

miRNA	Regulation	Region	Infection or Habit	Ref.
miR-101	Up	HNSCC	Alcohol	[148]
miR-107	Down	Oropharyngeal SCC	HPV	[134]
miR-1266	Up	HNSCC	Alcohol	[148]
miR-133a-3p	Down	Oropharyngeal SCC	Smoking	[144]
miR-139-5p	Down	HNSCC	HPV	[133]
miR-142-5p	Down	HNSCC	HPV	[132]
miR-143	Down	HNSCC	HPV	[133]
miR-145	Down	HNSCC	HPV	[133]
miR-145	Down	Oral Fibroblast	Smoking	[142]
miR-155	Down	HNSCC	HPV	[132]
miR-155	Up	Oropharyngeal	HPV	[135]
miR-155	Up	OSCC	Smoking	[141]
miR-155	Up	TSCC/BOTSCC	HPV	[138]
miR-15a	Up	HNSCC	HPV	[133]
miR-16	Up	HNSCC	HPV	[133]
miR-181a	Down	HNSCC	HPV	[132]
miR-181b	Down	HNSCC	HPV	[132]
miR-183	Up	Tongue Cancer	Alcohol	[150]
miR-18a	Down	Oropharyngeal SCC	HPV	[135]
miR-195	Down	HNSCC	HPV	[133]
miR-218	Down	HNSCC	HPV	[132]
miR-221	Down	HNSCC	HPV	[132]
miR-222	Down	HNSCC	HPV	[132]
miR-223	Down	Oropharyngeal SCC	HPV	[135]
miR-29a	Down	HNSCC	HPV	[132]
miR-30a	Up	HNSCC	Alcohol	[148]
miR-31	Down	Oropharyngeal SCC	HPV	[135]
miR-3164	Up	HNSCC	Alcohol	[148]
miR-3178	Up	HNSCC	Alcohol	[148]
miR-324-5p	Down	Oropharyngeal SCC	HPV	[134]
miR-33	Up	HNSCC	HPV	[132]
miR-34a	Up	OSCC	Alcohol	[74]
miR-34c-5p	Up	Laryngeal Epithelial Premalignant Lesions	Alcohol	[149]
miR-363	Up	HNSCC	HPV	[132]
miR-3690	Up	HNSCC	Alcohol	[148]
miR-381	Down	HNSCC	HPV	[133]
miR-497	Up	HNSCC	HPV	[132]
miR-497	Down	HNSCC	HPV	[133]
miR-499a	Up	HNSCC	Smoking	[145]
miR-574-3p	Down	HNSCC	HPV	[133]
miR-675	Up	HNSCC	Alcohol	[148]
miR-9	Up	Oropharyngeal SCC	HPV	[135]
miR-9	Up	OSCC, Oropharyngeal SCC	HPV	[137]
miR-934	Up	HNSCC	Alcohol	[148]

## Abbreviations

miRNA	MicroRNA
HNSCC	Head and neck squamous cell carcinoma
HNC	Head and neck cancer
LCa	Laryngeal cancer
qRT-PCR	Quantitative real-time PCR
RISC	RNA-induced silencing complex
OSCC	Oral squamous cell cancer
SACC	Salivary adenoid cystic carcinoma
MSSCC	Maxillary sinus squamous cell carcinoma
EMT	Epithelial-mesenchymal transition
OTSCC	Oral tongue squamous cell carcinoma
NPC	Nasopharyngeal carcinoma
TSCC	Tongue squamous cell carcinoma
SN-SCC	Sinonasal squamous cell carcinomas
HSCC	Hypopharyngeal squamous cell carcinoma
EBV	Epstein–Barr virus
LRC	Locoregional control
MDR	Multiple drug resistance
ACC	Adenoid cystic carcinoma
Pim-1	Moloney murine leukemia virus 1
AUC	Area under curve
PUMA	p53-upregulated modulator of apoptosis
ROC	Receiver operating characteristic
HPV	Human papillomavirus
SCC	Squamous cell carcinoma
TSCC/BOTSCC	Tonsillar and base of tongue cancer
